# Classification Performance and Feature Space Characteristics in Individuals With Upper Limb Loss Using Sonomyography

**DOI:** 10.1109/JTEHM.2022.3140973

**Published:** 2022-01-06

**Authors:** Susannah Engdahl, Ananya Dhawan, Ahmed Bashatah, Guoqing Diao, Biswarup Mukherjee, Brian Monroe, Rahsaan Holley, Siddhartha Sikdar

**Affiliations:** Department of BioengineeringGeorge Mason University 3298 Fairfax VA 20030 USA; Department of Biostatistics and BioinformaticsThe George Washington University 8367 Washington DC 20052 USA; Hanger Clinic 517125 Laurel MD 20707 USA; MedStar National Rehabilitation Hospital Washington DC 20010 USA

**Keywords:** Upper limb, pre-prosthetic training, prosthesis control, sonomyography, feature space

## Abstract

*Objective*: Sonomyography, or ultrasound-based sensing of muscle deformation, is an emerging modality for upper limb prosthesis control. Although prior studies have shown that individuals with upper limb loss can achieve successful motion classification with sonomyography, it is important to better understand the time-course over which proficiency develops. In this study, we characterized user performance during their initial and subsequent exposures to sonomyography. *Method*: Ultrasound images corresponding to a series of hand gestures were collected from individuals with transradial limb loss under three scenarios: during their initial exposure to sonomyography (Experiment 1), during a subsequent exposure to sonomyography where they were provided biofeedback as part of a training protocol (Experiment 2), and during testing sessions held on different days (Experiment 3). User performance was characterized by offline classification accuracy, as well as metrics describing the consistency and separability of the sonomyography signal patterns in feature space. *Results*: Classification accuracy was high during initial exposure to sonomyography (96.2 ± 5.9%) and did not systematically change with the provision of biofeedback or on different days. Despite this stable classification performance, some of the feature space metrics changed. *Conclusions*: User performance was strong upon their initial exposure to sonomyography and did not improve with subsequent exposure. *Clinical Impact*: Prosthetists may be able to quickly assess if a patient will be successful with sonomyography without submitting them to an extensive training protocol, leading to earlier socket fabrication and delivery.

## I. Introduction

Despite the enormous investment of resources in the development of new multi-articulated upper limb prosthetics, a large proportion of individuals with upper limb loss discontinue use of their prosthesis [1]–[3]. Users often experience dissatisfaction with the function and control of their prosthesis [4], [5], so it is crucial that they receive training to mitigate the challenges associated with using the device [6], [7]. Although training has been correlated with increased prosthesis use [8] and satisfaction [9], patient access to rehabilitation and prosthetic services in the United States is frequently limited [10]. There is also a scarcity of clinicians who specialize in treating upper limb loss [11] and possess the specialized knowledge required to train patients on effective prosthesis use. The difficulty of learning to use a prosthesis may be apparent given certain limitations in the predominant method for sensing and decoding user intent, surface electromyography (EMG). EMG is limited by poor amplitude resolution and low signal-to-noise ratio, especially with dry electrodes used in prosthesis sockets [12], [13]. There is also low specificity between muscles due to cross-talk and co-activation, especially for deep-seated muscle groups in the forearm that are responsible for finger movement [14]–[17]. Consequently, multi-articulated hands tend to rely on direct control strategies for opening and closing the terminal device in which EMG signals are recorded from an agonist-antagonist muscle pair. Other methods for controlling multi-articulated prosthetic hands rely on pattern recognition to decode user intent from EMG signal patterns. Although pattern recognition algorithms enable successful real-time grasp classification [18]–[20] and allow for control of a prosthetic hand during real-world functional tasks [21]–[24], users and therapists both report that extended periods of training are typically necessary to achieve stable performance [25], [26].

Given the challenges related to training and reliably decoding user intent with surface EMG, some researchers are pursuing an alternative approach called sonomyography (SMG), which uses ultrasound to sense muscle deformations in the residual limb. Although surface EMG provides temporal information about muscle deformation based on electrical features, ultrasound permits both temporal and spatial characterization of the deformation based on the acquired images. This spatiotemporal information is accessible even for muscles in deep-seated compartments, thus avoiding the problem of low specificity. Numerous prior studies have demonstrated clear potential for the use of SMG in controlling a prosthesis or other human-machine interface [27]–[34]. Our own work has shown that SMG is capable of accurately classifying motor intent for individuals without upper limb loss [35], [36] and individuals with upper limb loss [37], [38] in both offline and real-time settings. In particular, we have demonstrated an offline classification accuracy of 96% for five grasps in individuals with upper limb loss [38]. While these early studies have demonstrated robust classification performance is possible with SMG, the primary clinical benefits have not yet been established. To successfully translate SMG to clinical practice, it is important to better understand the clinical need that SMG can fill. In particular, it remains unknown how quickly prosthesis users can learn to use SMG and repeatably isolate control signals. Our study is focused on investigating this question.

Regardless of the specific prosthesis control strategy, the process by which naïve patients learn to use their device encompasses multiple stages [39], including pre-prosthetic and prosthetic training. The pre-prosthetic training stage involves learning how to generate the requisite control signals for operating a prosthesis, but is usually accomplished without the use of a physical prosthesis. Users are provided various sources of biofeedback, often within a virtual environment, to help them understand what happens physiologically when they perform a movement and how to modulate that activity for prosthetic control. For example, they might view a real-time representation of the EMG signal to demonstrate how muscle contractions are linked to electrical activity. This functionality is available in several commercial products, such as Ottobock MyoBoy, Ottobock Myo Plus, and Coapt Complete ControlRoom. The prosthetic training stage includes performing functional tasks with a physical prosthesis to become proficient using it in real-world settings. In this paper, we will restrict our discussion to the pre-prosthetic training stage.

During pre-prosthetic training for EMG pattern recognition, the user must learn to produce a specialized set of EMG patterns that are sufficiently consistent and separable from each other to permit accurate gesture classification. This can be difficult since people generally do not have experience modulating EMG signal amplitudes [40]. Individuals with limb loss may be further disadvantaged by motor cortex reorganization following amputation [41], as well as muscle atrophy due to disuse of the residual limb and/or increased reliance on the intact limb [42]. Given these difficulties, it is unsurprising that first attempts to use pattern recognition are often error-prone. For example, one study reported an average initial classification accuracy for individuals with transradial limb loss to be 77.5% for nine motion classes [43]. Training over the course of multiple sessions or days appears to mitigate some of these errors for individuals with and without limb loss, regardless of whether feedback on their performance is provided [43]–[46]. These improvements are credited to changes in the EMG signal patterns such that they become more consistent and/or separable, although the correlation between performance and EMG pattern characteristics is complex and not yet fully understood [46]–[49].

Beyond the primary purpose of helping patients learn how to use a prosthesis, pre-prosthetic training serves an important clinical function by helping prosthetists to understand whether their patients are cognitively and physiologically capable of using a particular control modality. Fabricating and fitting an upper limb prosthesis is time-consuming and expensive, especially if multi-articulated prosthetic hands are included, so prosthetists must ensure patients are suited to the control modality prior to beginning the process [50]. In the case of EMG pattern recognition, patients must be able to produce consistent and separable EMG signal patterns. However, it can be difficult to demonstrate this ability quickly given the prolonged time period needed to develop proficiency, creating a burden on both the prosthetist and patient. Reduced pre-prosthetic training times and quicker assessment of a prospective user’s ability to generate consistent and separable control signals may help improve a patient’s ease of access to care.

In this study, we investigated whether users can quickly and repeatably isolate SMG control signals during pre-prosthetic training. We characterized user performance during their initial and subsequent exposures to SMG in order to better understand the time-course over which proficiency develops. Performance was characterized by offline classification accuracy, as well as metrics describing the consistency and separability of SMG signal patterns in feature space. We asked three questions: 1) What is the performance during initial exposure to SMG? 2) Is biofeedback useful in helping users change their performance? 3) Is performance repeatable across multiple exposures to SMG?

## II. Methods

### A. Subjects

We recruited eight participants, including seven individuals with transradial limb amputation and one individual with congenital limb absence (Table 1). These individuals reported using myoelectric prostheses in their daily lives, but were naïve to the use of SMG prior to beginning the study. All individuals provided written informed consent prior to participating in this study, which was approved by the Institutional Review Boards at George Mason University (#492701, approved Oct. 24, 2013) and MedStar National Rehabilitation Network (#2016-173, approved Sept. 21, 2016).

**TABLE 1:** Participant Characteristics

ID	Sex	Years since amputation	Prior prosthesis experience^a^	Affected limb	Experiment 1	Experiment 2	Experiment 3^c^
Am1	M	46	Direct control (experienced)	Right	X	X	X (Session 1 – 2 datasets, Session 2 – 11 datasets)
Am2	M	Congenital	unknown	Left	X		
Am3	M	50	Direct control (experienced), Patten recognition (novice)	Left	X	X	X (Session 1 – 6 datasets, Session 2 – 11 datasets)
Am4	M	7.5	Patten recognition (experienced)	Left	X		
Am5	M	1.5	Direct control (novice), Patten recognition (novice)	Right^b^	X	X	X (Session 1 – 11 datasets, Session 2 – 4 datasets)
Am6	M	1	Direct control (novice), Patten recognition (novice)	Right	X		
Am7	M	1	Direct control (novice)	Left	X	X	
Am8	F	12	Direct control (experienced)	Right^b^	X	X	

^a^ Novice indicates less than two years of experience

^b^ Participant was affected bilaterally

^c^ Different numbers of datasets were collected during Experiment 3 due to variation in the participants’ availability and stamina

### B. Protocol

We conducted three different experiments to evaluate our research questions, with some individuals participating in only a subset of the experiments depending on their availability.

#### 1) Experiment 1

All eight participants were included in Experiment 1. Data collection was performed using a clinical ultrasound system (Terason uSmart 3200T, Terason, Burlington, MA). A low-profile, high-frequency, linear 16HL7 ultrasound transducer was positioned on the volar aspect of participants’ residual limb using a stretchable fabric cuff such that muscle deformations associated with all individual phantom finger movements were visually identifiable on the ultrasound images. Ultrasound image sequences were acquired and transferred to a PC in real-time using a USB-based video grabber (DVI2USB 3.0, Epiphan Systems, Inc.). The captured screen was then downscaled to 100 $\times140$ pixels to include only the relevant ultrasound image. The acquired image frames were processed in MATLAB (MathWorks, Natick, MA) using custom algorithms.

To create a dataset for training the classifier, participants performed repeated iterations of one motion from a set of motions that they felt were intuitive to perform. Am2 and Am3 performed power grasp, wrist pronation, thumb flexion, and index finger flexion. Am6 performed wrist pronation and supination, wrist flexion and extension, power grasp, ulnar deviation, and thumb flexion. The other participants performed power grasp, wrist pronation, key grasp, tripod, and index point.

Starting from a resting position, participants followed an auditory cue and moved towards the end state of the desired motion over the course of one second, held the end state position for one second, moved back to rest over the course of one second, and remained at rest for one second. After they repeated this process five times in succession, we extracted the ultrasound image frames corresponding to the motion end state and rest. This process was repeated until all five motions were included in the dataset. Once the dataset was completed, we performed leave-one-out cross-validation with a modified 1-nearest-neighbor classifier that used Pearson’s correlation coefficient as a similarity measure [36]. Our modified classifier averages the similarity measurements by class and selects the most similar class instead of selecting the most similar individual image.

#### 2) Experiment 2

A subset of five participants was included in Experiment 2. Data collection procedures were identical to Experiment 1, except that participants instead created a series of datasets across two different phases of data collection. Both phases occurred during a single day. Additionally, all participants performed the same five motions in Experiment 2 (power grasp, wrist pronation, key grasp, tripod, index point).

During the first phase (*baseline*), participants were asked to create three datasets to establish their baseline performance for that day. We did not provide any feedback to participants about their performance during this phase.

During the second phase (*feedback*), participants were asked to create three datasets while receiving verbal and visual biofeedback about their performance. Specifically, participants were allowed to view the ultrasound images in real-time so that they could understand their residual limb anatomy and how their muscles deformed when attempting each grasp. They were also asked to describe their sensations of phantom hand movement associated with each grasp and to demonstrate the movement using their intact hand. Based on their explanation, we offered suggestions on how they could make their movements more separable.

**FIGURE 1.**
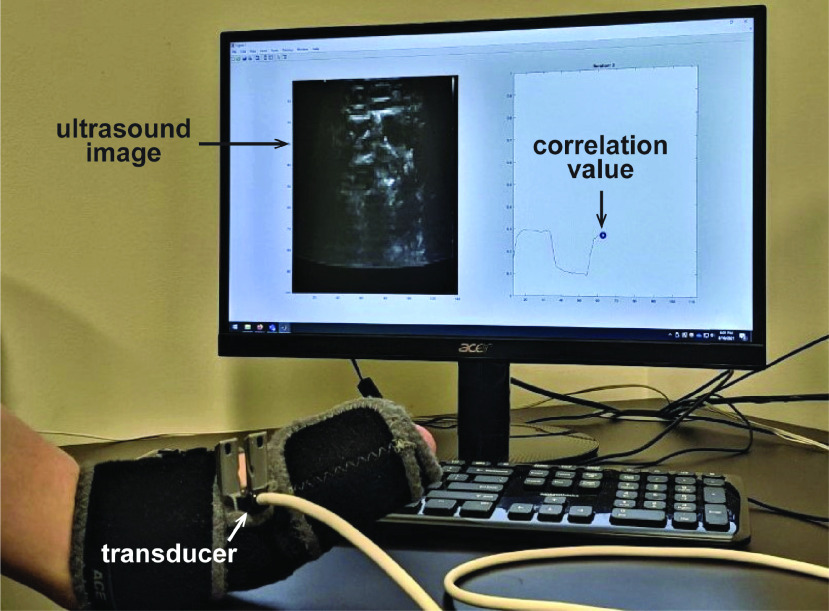
Visual display used to provide feedback to participants during Experiment 2. The Pearson correlation between the current ultrasound image and the first image recorded in the sequence is indicated by the blue circle. High correlation values indicate that current image is dissimilar from rest, while low values indicate similarity to rest. Correlation values for previous images remained on the display (red line) to help participants achieve consistency in the muscle deformation pattern.

Participants were also given a visual cue to help them monitor the consistency in their muscle deformation patterns (Fig. 1). For each movement sequence, we calculated the Pearson correlation between the first ultrasound image frame (corresponding to a rest state) and the incoming image. The correlation value was inverted and graphically displayed in real-time such that high values indicated dissimilarity from rest (i.e., motion end state) and low values indicated similarity to rest. A “plateau” of high correlation values during the one-second hold period for each motion end state would indicate that the muscle deformation pattern was similar between successive images. Thus, while this visual display does not reveal which individual muscles are being contracted, it can help participants achieve consistency in the overall deformation pattern.

Finally, participants were told the results of the cross-validation after creating each dataset and were shown the associated confusion matrix to help them understand the source of any errors. They were also given suggestions on how they could alter their movements to try and improve the classification accuracy. For example, power grasp and key grasps were occasionally confused because these movements are fairly similar. In this case, participants might be instructed to increase thumb adduction during key grasp to further differentiate the motion from power grasp.

Although best practices for teaching SMG have not yet been established, we believe that the general principles used for teaching EMG pattern recognition should be applicable. Thus, most of the strategies we used for delivering biofeedback have been recommended in existing literature. This includes asking the user to move the phantom limb or intact hand to mirror the intended grasp [39], [51], showing the user raw signals to demonstrate that different grasps are associated with different muscle activation patterns [39], [51], offering suggestions on how to make movements more separable [51], and showing the user a confusion matrix to demonstrate which grasps were confused [51]. While the Pearson correlation display was a unique approach to this study, other studies have used visual displays to help users develop consistency in their muscle activation (e.g., [48]). It is also important to note that we did not follow a rigorously structured protocol for delivering identical feedback to every participant, but this format is similar to what is done in clinical settings for patients learning EMG pattern recognition. The prosthetist or therapist may use the same general techniques for all patients, such providing a visual display to show how different movements produce distinct muscle activity patterns or asking patients to consider the movement of their phantom hands. However, training is conducted under the clinician’s supervision so that specific instructions can be customized for each patient based on their residual limb anatomy and rehabilitation goals [39], [47], [51]. Similarly, we provided the same general sources of feedback to our participants but customized the specific instructions when necessary.

#### 3) Experiment 3

A subset of three participants was included Experiment 3. Data collection procedures were identical to Experiments 1, except that participants created a series of datasets across two data collection sessions occurring on separate days. All participants performed the full set of five motions in Experiment 2 (power grasp, wrist pronation, key grasp, tripod, index point). We intended to collect as many datasets as possible from each participant depending on their availability and stamina. Consequently, we obtained differing numbers of datasets across participants and sessions (Table 1).

### C. Data Analysis

The primary outcome metric was cross-validation accuracy (1), defined as the percent of data correctly classified during the leave-one out validation process for a given dataset i:

\begin{equation*} {CA}_{i}=100\ast \frac {P_{correct_{i}}}{P_{total_{i}}},\tag{1}\end{equation*}

where $P_{correct_{i}}$ is the correct number of predictions by the closest-class classifier and $P_{total_{i}}$ is the total number of predictions (i.e., the total number of datapoints).

Cross-validation accuracy is a combined measurement of the user’s ability to perform a motion and the classifier’s ability to label individual motion performances. Since user performance and classifier performance are inherently linked in this metric, it is possible that a user’s performance could change over time without affecting the cross-validation accuracy. For example, a user may perform the tripod grasp with very little variation for a given dataset, resulting in a high cross-validation accuracy. On the next dataset, they may perform the grasp with two different variations having slightly different levels of middle finger flexion. As long as the closest identified class for each of the variations is still tripod, the cross-validation accuracy would be unaffected. Therefore, we wanted to more appropriately understand the changes in user performance independent of the classifier performance.

To accomplish this goal, we performed a dimensionality reduction on all individual datasets collected from the participants during each experiment. The datasets contained all ultrasound images that were recorded for each grasp. Every 100 $\times140$ pixel image in the dataset was represented as points in 14,000-dimensional space such that each pixel in the image corresponded to an axis in the high dimensional space. This high dimensional space was then reduced to five dimensions through principal components analysis. Next, we defined point clusters in five-dimensional space such that each cluster was comprised of all the points in an associated motion class. We then utilized several metrics to describe the characteristics of these clusters in SMG feature space, including Within-class Distance (WD), Inter-class Distance Nearest Neighbor (IDNN), Inter-class Distance All Neighbors (IDAN), Most Separable Dimension (MSD), and Mean Semi-Principal Axis (MSA) [46]. These metrics characterize the clusters’ consistency or separability. If a participant’s performance of a given motion becomes more similar to other performances of the same motion, the points in that motion cluster move closer together and the consistency increases. If a participant’s performance of a given motion becomes more distinct from the performances of another motion, the clusters move further apart and the separability increases. A more detailed explanation of each metric is provided below.

#### 1) Within-Class Distance

WD is a measure of consistency between all five repetitions of the same motion

\begin{equation*} {WD}_{j}=\sum \nolimits _{r=1}^{5} \sum \nolimits _{k=1}^{5} \frac {dist_{kj}^{rj}\ast {dist}_{rj}^{kj}}{dist_{kj}^{rj}+{dist}_{rj}^{kj}}.\tag{2}\end{equation*}

Here, ${dist}_{kj}^{rj}$ is half the Mahalanobis distance in feature space between repetitions $r$ and $k$ of motion $j$, and ${dist}_{rj}^{kj}$ is half the Mahalanobis distance in feature space between repetitions $k$ and $r$ of motion $j$

\begin{align*} {dist}_{kj}^{rj}=&\frac {1}{2}\sqrt {\left ({\left ({\mu _{Trj}-\mu _{Tkj} }\right)^{T}\ast S_{Trj}^{-1}\ast \left ({\mu _{Trj}-\mu _{Tkj} }\right) }\right)} \tag{3}\\ {dist}_{rj}^{kj}=&\frac {1}{2}\sqrt { \left ({\left ({\mu _{Tkj}-\mu _{Trj} }\right)^{T}\ast S_{Tkj}^{-1}\ast \left ({\mu _{Tkj}-\mu _{Trj} }\right) }\right)}\tag{4}\end{align*}

where $\mu _{Trj}$ and $\mu _{Tkj}$ represent the feature vectors from repetitions $r$ and $k$, respectively. $S_{Trj}$ and $S_{Trj}$ are the covariances from repetitions $r$ and $k$, respectively. The total WD for each participant is defined as the mean WD over all $n$ motions:

\begin{equation*} {WD}_{total}=\frac {1}{n}\sum \nolimits _{j=1}^{n} {WD}_{j}.\tag{5}\end{equation*}

#### 2) Inter-Class Distance Nearest Neighbor

IDNN is a measure of separability between different motions

\begin{equation*} {IDNN}_{i}=\mathop {\mathrm {min}}_{i=1,\ldots,j-1,j+1,\ldots,n}\frac {dist_{j}^{i}\ast {dist}_{i}^{j}}{dist_{j}^{i}+{dist}_{i}^{j}}\tag{6}\end{equation*}

such that only the distance to a motion’s nearest neighbor is included. Here, ${dist}_{j}^{i}$ is half the Mahalanobis distance in feature space between motions $i$ and $j$, and ${dist}_{i}^{j}$ is half the Mahalanobis distance in feature space between motions $j$ and $i$

\begin{align*} {dist}_{j}^{i}=&\frac {1}{2}\sqrt {\left ({\left ({\mu _{Ti}-\mu _{Tj} }\right)^{T}\ast S_{Ti}^{-1}\ast \left ({\mu _{Ti}-\mu _{Tj} }\right) }\right)} \tag{7}\\ {dist}_{i}^{j}=&\frac {1}{2}\sqrt { \left ({\left ({\mu _{Tj}-\mu _{Ti} }\right)^{T}\ast S_{Tj}^{-1}\ast \left ({\mu _{Tj}-\mu _{Tj} }\right) }\right)}\tag{8}\end{align*}

where $\mu _{Ti}$ and $\mu _{Tj}$ represent the feature vectors from motions $i$ and $j$, respectively. $S_{Tj}$ and $S_{Tj}$ are the covariances from motions $i$ and $j$, respectively. The total IDNN for each participant is defined as the mean IDNN over all $n$ motions:

\begin{equation*} {IDNN}_{total}=\frac {1}{n}\sum \nolimits _{j=1}^{n} {IDNN}_{j}.\tag{9}\end{equation*}

#### 3) Inter-Class Distance All NEIGHBORS

IDAN is a measure of separability between different motions and is similar to IDNN, except that distances to all neighbors are included

\begin{equation*} {IDAN}_{i}=\sum \nolimits _{j=1}^{n} \frac {dist_{j}^{i}\ast {dist}_{i}^{j}}{dist_{j}^{i}+{dist}_{i}^{j}}.\tag{10}\end{equation*}

The total IDAN for each participant is defined as the mean IDAN over all $n$ motions:

\begin{equation*} {IDNN}_{total}=\frac {1}{n}\sum \nolimits _{j=1}^{n} {IDAN}_{j}.\tag{11}\end{equation*}

#### 4) Most Separable Dimension

MSD is similar to IDNN, except that only one of the five dimensions is used to determine the nearest neighbor. In this case, the dimension with the largest separability is chosen

\begin{equation*} {MSD}_{i}=\mathop {\mathrm {min}}_{i=1,\ldots,j-1,j+1,\ldots,n}\left ({\mathop {\mathrm {max}}_{d=1,\ldots,5}\frac {dist_{j}^{i}\ast {dist}_{i}^{j}}{dist_{j}^{i}+{dist}_{i}^{j}} }\right)\tag{12}\end{equation*}

The total MSD for each participant is defined as the mean MSD over all $n$ motions:

\begin{equation*} {MSD}_{total}=\frac {1}{n}\sum \nolimits _{j=1}^{n} {MSD}_{j}.\tag{13}\end{equation*}

#### 5) Mean Semi-Principal Axis

MSA is a measure of variability for a given motion and is calculated as a geometric mean

\begin{equation*} {MSA}_{k}=\left ({\prod \nolimits _{k=1}^{5} a_{k} }\right)^{\frac {1}{5}}\tag{14}\end{equation*}

where $a_{k}$ is the semi-principal axes of the hyperellipsoids. The total MSA for each participant is defined as the mean MSA over all $n$ motions:

\begin{equation*} {MSA}_{total}=\frac {1}{n}\sum \nolimits _{j=1}^{n} {MSA}_{j}.\tag{15}\end{equation*}

### D. Statistical Analysis

For Experiment 2, we compared each outcome metric (cross-validation accuracy, WD, IDNN, IDAN, MSD, MSA) from the *baseline* phase to the means from the *feedback* phase using the following linear mixed model

\begin{equation*} Y_{ij}=\beta _{0}+b_{i}+\beta _{1}X_{ij}+\epsilon _{ij},\tag{16}\end{equation*}

where $Y_{ij}$ is the outcome metric for the $i$th subject at the $j$th measurement, $X_{ij}$ is a dichotomous variable with value 0 for the *baseline* phase and 1 for the *feedback* phase, $\epsilon _{ij}$ is the residual error, and $b_{i}$ is a random intercept accounting for within-subject correlations among repeated measures. Both $b_{i}$ and $\epsilon _{ij}$ are assumed to be normally distributed and independent. The baseline phase is treated as the reference level. To account for the small sample size and potential violation of the model assumptions, we used the permutation test [52] to assess significance ( $\alpha = 0$.05). One-sided p-values were based on 1000 permuted samples.

To assess whether there was a change over time for the outcomes, we fit the same model as (16) but replaced the dichotomous variable $X_{ij}$ with the normalized time from the first measurement in the *baseline* phase. It is worth noting that it is not appropriate to include both normalized time and the dichotomous feedback variable in the linear mixed models since they are highly correlated with a Pearson correlation coefficient 0.923 (p <1.0e-12).

To evaluate the effect of the feature space metrics on cross-validation accuracy, we fit the following linear mixed model

\begin{align*}&\hspace {-0.7pc}Y_{ij}=\beta _{0}+b_{i}+\beta _{1}X_{ij1}+\beta _{2}{WD}_{ij}+\beta _{3}{IDNN}_{ij}+\beta _{4}{IDAN}_{ij} \\&\qquad\qquad\qquad\qquad\qquad+\,\beta _{5}{MSD}_{ij}+\beta _{6}{MSA}_{ij}+\epsilon _{ij},\tag{17}\end{align*}

where $X_{ij}$ is either a dichotomous variable with value 0 for the *baseline* phase and 1 for the *feedback* phase or the normalized time, and ${WD}_{ij},{IDNN}_{ij},{IDAN}_{ij},{MSD}_{ij},{MSA}_{ij}$ are the feature space metrics. The baseline phase is treated as the reference level. Two-sided p-values were based on 1000 permuted samples.

For Experiment 3, we compared each outcome metric between sessions 1 and 2. We fit the following linear mixed model

\begin{equation*} Y_{ij}=\beta _{0}+b_{i}+\beta _{1}X_{ij1}+\beta _{2}X_{ij2}+\epsilon _{ij},\tag{18}\end{equation*}

where $X_{ij1}$ is the normalized time from the first measurement in the *baseline* phase, and $X_{ij2}$ takes value 0 for session 1 and 1 for session 2. Compared to (16), this model includes two covariates. Since the phase variable and the normalized time are highly correlated and there were very few observations for some phases in some subjects, we choose to compare the mean outcomes between session 1 and session 2 while controlling for the confounding effects of the normalized time. To account for the small sample size and potential violation of the model assumptions, we used the permutation test [52] to assess significance ( $\alpha = 0$.05). Two-sided p-values were based on 1000 permuted samples.

To evaluate the effect of the feature space metrics on cross-validation accuracy, we fit the following linear mixed model

\begin{align*}&\hspace {-0.5pc}Y_{ij}=\beta _{0}+b_{i}+\beta _{1}X_{ij1}+\beta _{2}X_{ij2}+\beta _{3}{WD}_{ij}+\beta _{4}{IDNN}_{ij} \\&\qquad\qquad\qquad+\,\beta _{5}{IDAN}_{ij}+\beta _{6}{MSD}_{ij}+\beta _{7}{MSA}_{ij}+\epsilon _{ij},\tag{19}\end{align*}

where $X_{ij1}$ is the normalized time from the first measurement in the *baseline* phase, and $X_{ij2}$ takes value 0 for session 1 and 1 for session 2. Two-sided p-values were based on 1000 permuted samples.

## III. Results

### A. Experiment 1

**FIGURE 2.**
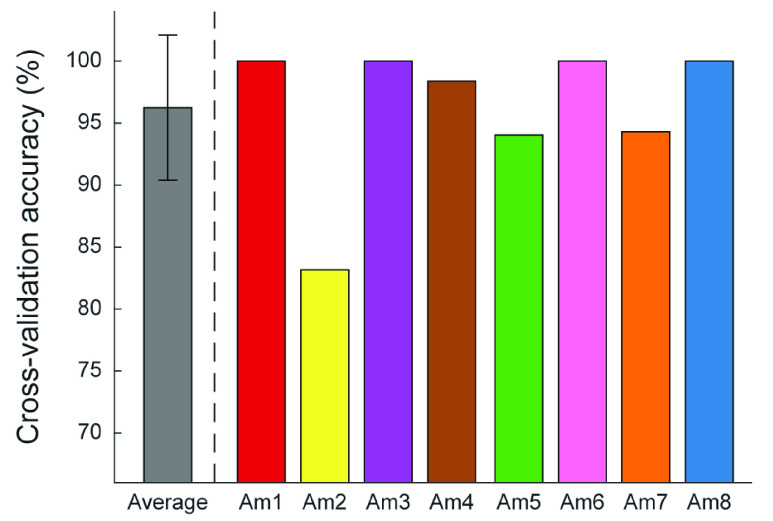
Between-subject average (grey bar) and per-subject (colored bars) cross-validation accuracy. Error bar represents standard deviation.

The average cross-validation accuracy across subjects was 96.2 ± 5.9%, with a range of 83.2% to 100% (Fig. 2). The SMG feature space metrics were moderately correlated with cross-validation accuracy ( $0.47\le \vert \text{r}\vert \le 0.76$; Supplementary Fig. S1), although none of the correlations were significant except for WD (p = 0.03). Grasp-specific metrics did not follow a consistent trend, but wrist pronation and/or supination often had the lowest MSA and highest IDNN, IDAN, and MSD among all grasps collected from individual participants (Supplementary Figs. S2-S6).

### B. Experiment 2

**FIGURE 3.**
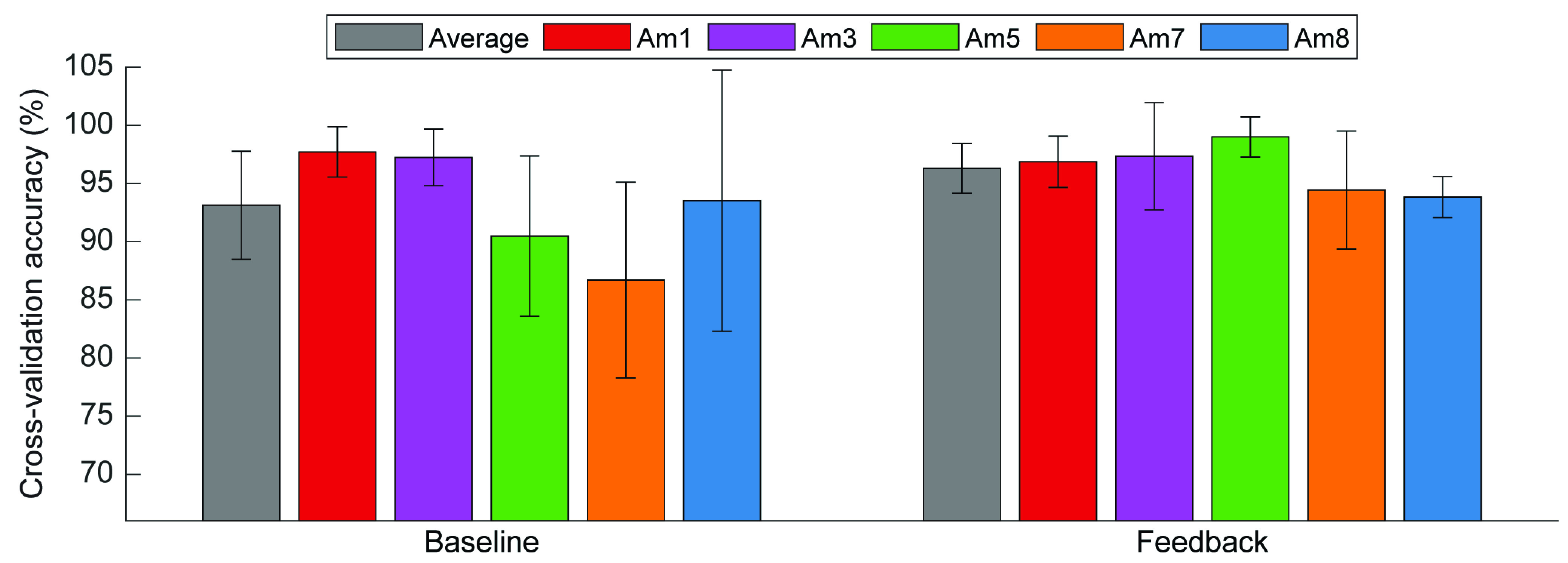
Average between-subject (grey bars) and within-subject (colored bars) cross-validation accuracy for each phase. Error bars represent standard deviation.

Cross-validation accuracy exceeded 77% for all 30 datasets collected across participants. Furthermore, 19 of these datasets had a cross-validation accuracy of at least 95%. The average cross-validation accuracy was 93% or higher for both phases (*baseline*: 93.1 ± 4.6%; *feedback*: 96.3 ± 2.1%; Fig. 3). The average elapsed collection time was 69 ± 47 minutes (Supplementary Fig. S7), although this value is elevated by the unusually long testing time for Am7 (151 minutes). The elapsed time was much more consistent across the remaining subjects (49 ± 14 minutes). There was no significant effect of phase (p = 0.08) or elapsed time (p = 0.141) on cross-validation accuracy (Supplementary Tables S1 and S2).

Although overall cross-validation accuracy for all five grasps was generally high, the accuracy values for individual grasps reveal that there was occasional misclassification. However, visual inspection of the misclassification rates across all six datasets for each participant shows no obvious patterns over time (Supplementary Fig. S8).

There was a significant increase in IDNN (p = 0.012) and IDAN (p = 0.012) from the *baseline* phase to the *feedback* phase. Similarly, there was a significant increase in IDNN (p = 0.032) and IDAN (p = 0.018) over time. There were no significant changes in the other feature space metrics between phases or over time (Supplementary Tables S1 and S2). The overall range for each metric varied slightly between participants (Supplementary Fig. S9). Of the five feature space metrics, only MSD had a marginally significant effect on cross-validation accuracy (Supplementary Table S3).

### C. Experiment 3

**FIGURE 4.**
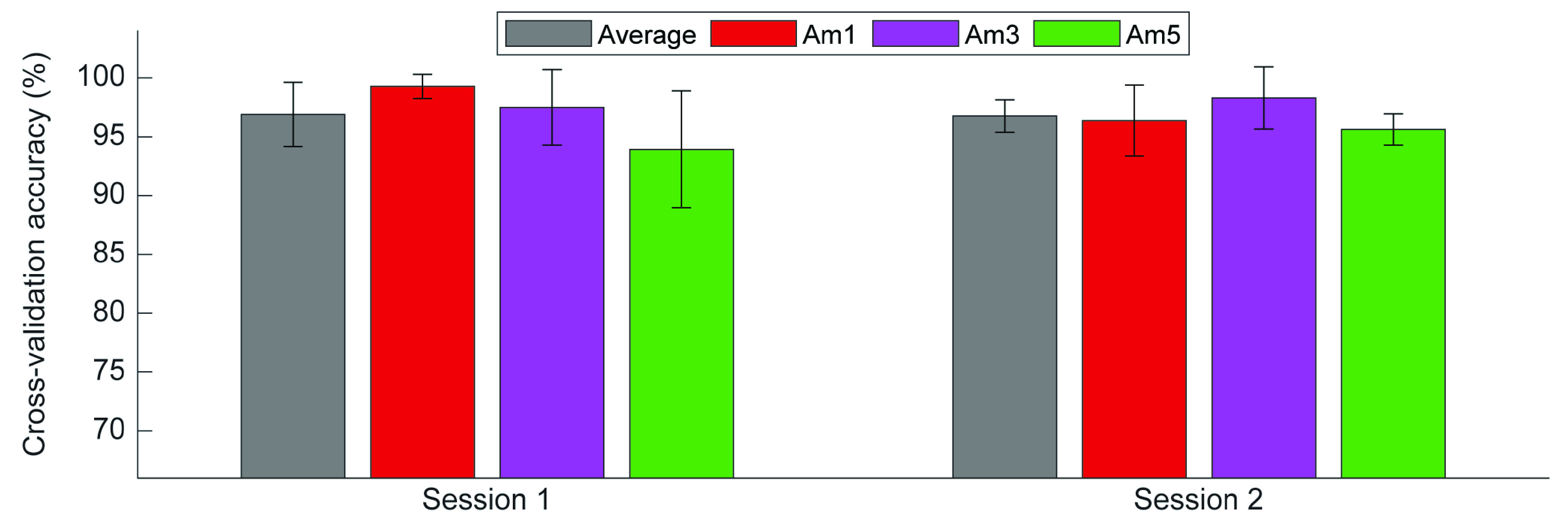
Average between-subjects (grey bars) and within-subject (colored bars) cross-validation accuracy for each session. Error bars represent standard deviation.

The cross-validation accuracy did not change significantly between sessions (session 1: 96.9 ± 2.7%, session 2: 96.7 ± 1.4%; p = 0.586; Fig. 4). There was a significant increase in IDNN (p = 0.013), IDAN (p = 0.038), and MSA (p < 0.001) between sessions, but no change in the other feature space metrics (Supplementary Table S4). The overall range for each metric varied slightly between participants (Supplementary Figs. S10-S12). None of the five feature space metrics had a significant effect on cross-validation accuracy (Supplementary Table S5).

## IV. Discussion

The primary purpose of this study was to characterize user performance during their first and subsequent exposures to SMG. We demonstrated that participants’ classification performance during their first experience with SMG was very strong (96.2 ± 5.9%). Am2 had the poorest performance of all the participants (83.2%), which may be a consequence of having congenital limb absence. It is possible that Am2 had a limited phantom hand sensations or proprioceptive sense in his residual limb, making it challenging to perform the hand gestures. Nonetheless, his performance was still stronger than some first-time users of EMG pattern recognition, who have achieved classification accuracies as low as 77.5% for nine motion classes [43]. Am2’s performance may have improved with additional practice, but we could not test this hypothesis because he was unable to participate in Experiment 2.

We found that most participants were able to generate the requisite control signals on their first try, even without biofeedback. There may be different explanations, but one possibility is that SMG provides both spatial and temporal information within the ultrasound image sequence about the user’s muscle deformation. This means the problem of low specificity between muscles due to cross-talk and co-activation is avoided. In turn, users are able to generate muscle contractions that are congruent with the intended grasp, without needing to modify the contraction to make it more distinct from other grasps. These results indicate that one important clinical benefit of SMG is to enable prosthetists to quickly assess a prospective user’s ability to generate consistent and separable control signals. This assessment may allow the prosthetist to determine an appropriate treatment plan without requiring the user to go through a lengthy pre-prosthetic training phase.

Although our participants’ classification performance was strong even upon their first exposure to SMG, we asked whether their performance could improve further. In fact, their classification performance did not systematically change with the provision of biofeedback or between different data collection sessions. Nonetheless, it should be acknowledged that we cannot fully distinguish between the effects of repetitive practice and provision of biofeedback on classification performance during Experiment 2. Inclusion of a control group that underwent repeated exposures to SMG without biofeedback could have clarified whether these factors are dissociable, but it was infeasible to recruit an additional set of participants with limb loss. Another possibility would be to include an interaction term in the linear mixed models, but we chose not to do this because of the small sample size. However, visual inspection of the results leads us to believe that an interaction term would not have been significant even if it was included.

Most participants experienced small fluctuations in performance between datasets due to isolated misclassifications. These misclassifications may result from problems such as movement of the transducer with respect to the residual limb or minor variations in the muscle deformation patterns, which likely can be addressed with appropriate intervention (i.e., a more secure transducer mounting system or a classification algorithm less sensitive to variation than 1-nearest neighbor). While we do not believe the transient offline misclassifications observed in this study negate the potential viability of SMG as a control modality, additional study will be required to determine how significantly real-time misclassifications impact user performance in functional settings.

The lack of an online evaluation in this study is a limitation, as it is widely acknowledged that offline classification performance is not necessarily a definitive predictor of real-time control ability [53]. A variety of virtual testing environments, such as the Target Achievement Control test [54], have been developed to characterize user control strategies beyond simple classification accuracy and we are planning to include similar approaches in future studies. Testing with a physical prosthesis will also be necessary to explore the effect of factors like changes in arm position, sensor shifting, sweating, muscle fatigue, or changes in signal characteristics over time [55], which can degrade classification accuracy and require users to retrain the classifier after some period of use. We plan to characterize these issues in future work, as this is a crucial step in demonstrating the real-world viability of SMG.

We also noted inconsistencies in how the SMG feature space metrics changed with provision of biofeedback or between different data collection sessions. For both Experiments 2 and 3, distance between clusters increased according to IDNN and IDAN, suggesting that separability between grasps improved with biofeedback or between days. MSA also increased between days for Experiment 3, which indicates greater variability in how the grasps were performed.

Interestingly, the overall classification accuracy was stable despite these alterations in how participants performed the grasps. While it might be expected that classification accuracy would increase with greater intercluster distance and decrease with greater cluster variability, these relationships were not evident in our results. Given that the feature space metrics only explained 49.8-59.5% of the variability in classification accuracy (Supplemental Tables S3 and S5), this could mean that classification accuracy is related to other feature space patterns that were not quantified here. Alternatively, this finding may indicate that there are many ways to perform a grasp without sacrificing classification accuracy. The motor system is redundant and the same movement can be achieved through multiple muscle activation patterns. It is possible that our analysis method of using dimensionality reduction accounted for this redundancy during movement classification, but the differences in activation patterns persist in the other feature space metrics. Regardless, there is a precedent for this complex relationship between classification performance and feature space within the EMG literature, which has similarly reported discrepancies in how classification performance relates to EMG signal pattern characteristics [43]–[49]. However, most of these studies quantified real-time classification performance, rather than offline performance like in our study. Further comparison with the EMG literature should be withheld until real-time SMG performance can be studied.

Our finding that participants were immediately able to generate separable movements and could consistently repeat those movements represents a significant benefit of SMG. In comparison, people do not naturally have much experience with modulating EMG patterns [40], nor is it clear which EMG signal pattern characteristics are most relevant to classification performance [46]. It is therefore difficult to know how to effectively train users on EMG pattern recognition. As a result, pre-prosthetic training protocols can be lengthy, involving practice over multiple sessions or days with [43], [46] or without [44], [45] the provision of external feedback in order to improve classification performance. Developing training protocols and delineating relationships between signal pattern characteristics and classification performance appears to be less critical with SMG, as users seem capable of achieving successful classification without intervention.

Shortening the pre-prosthetic training phase with SMG could enable patients to devote more resources towards functional training with a physical prosthesis, which may still require involvement from a therapist. Furthermore, rapid pre-prosthetic training can simplify the process by which prosthetists evaluate whether patients are cognitively and physiologically able to use SMG. This evaluation could perhaps be completed in a single clinical visit, allowing prosthetists to proceed more quickly with socket fabrication if the patient has demonstrated an ability to use SMG or to recommend an alternative control modality if they are unable to use SMG.

A reduction in the amount of pre-prosthetic training may also help diminish barriers to prosthesis access in the United States, where few clinicians specialize in caring for people with upper limb loss or have experience with justifying a course of treatment to insurers [11]. For these reasons, it is perhaps unsurprising that one survey reported 35% of individuals with unilateral upper limb loss received no training of any kind and only 22% received more than 10 hours of training from a prosthetist or therapist [56]. Therapy is an essential component of the rehabilitation process and the receipt of training to use a first prosthesis has been associated with increased satisfaction [9]. Thus, we hypothesize that SMG may create a potential for increased satisfaction without the need for extensive involvement from a therapist. Experiencing an early sense of accomplishment from successfully learning the control strategy may also motivate users to continue practicing with the prosthesis and could reduce the likelihood that they abandon prosthesis use. We hope to investigate this hypothesis in future studies.

One limitation of this study is that sample size was small and the variability between participants may have reduced our ability to detect statistically significant results. While having a larger sample size would be useful, it should also be acknowledged that the characteristics of individuals with upper limb loss can be very heterogeneous and generalizing beyond the study sample should be done cautiously. As an example, Am7 had poorer classification performance in Experiment 2 compared to the other participants and required over twice as much time to create each dataset (Supplementary Fig. S7). Am7 had undergone amputation about one year prior to this study and was an extremely inexperienced myoelectric prosthesis user, having owned his prosthesis for only one week. He had significant muscle atrophy in his residual limb as a result of this disuse, which may have contributed to his difficulties with generating muscle contractions and relaxing his muscles to a “resting” position between repeated grasps. While these challenges wouldn’t necessarily preclude him from becoming a proficient SMG user, it is important to consider factors like this when assessing a patient’s potential for success with SMG. Thus, evaluations should be made on a case-by-case basis.

Additionally, we did not quantify the degree to which participants changed their performance after specific feedback was given. Since the initial classification accuracy was high, there was limited room for improvement. In future studies involving functional testing with a terminal device, we will quantify the specific ways in which feedback can improve performance.

Another limitation is that we utilized a commercially-available ultrasound imaging system with an array transducer. For translation of SMG technology to practical prosthesis sockets, we anticipate utilizing single-element transducers with low power electronics. Our previous work has indicated that the classification accuracy with sparse sensing is not compromised [57]. However, this result has yet to be validated in individuals with limb loss. We are currently developing fully-integrated prototype SMG systems and additional studies are planned in the future.

Finally, it should be noted that the reported classification accuracies were obtained using a 1-nearest neighbor classifier. We purposely utilized one of the simplest classifiers in an effort to decouple user performance from classifier performance. More sophisticated classifiers commonly used for EMG pattern recognition, such as linear discriminant analysis, are expected to provide improved classification accuracy.

## V. Conclusion

This study quantified classification performance and the associated SMG feature space for individuals with upper limb loss during their initial and subsequent exposures to SMG. We showed that participants could immediately achieve high motion classification accuracies and that their performance did not change with the provision of biofeedback or across multiple exposures to SMG. Some of the SMG feature space characteristics changed despite the stable classification accuracy, suggesting that these metrics do not fully predict classification performance. If SMG is deployed clinically in the future, the process of assessing patient suitability for SMG during pre-prosthetic training could be completed quickly, which ultimately may improve patient access to timely and appropriate care.
